# Left ventricular remodeling in children and young adults with aortic coarctation two decades after surgical repair

**DOI:** 10.1186/1532-429X-17-S1-Q96

**Published:** 2015-02-03

**Authors:** Ines Kristo, Philip Wegner, Inga Voges, Minh H Pham, Dominik Daniel Gabbert, Michael Jerosch-Herold, Ana C Andrade, Christopher Hart, Hans-Heiner Kramer, Carsten Rickers

**Affiliations:** 1 Department of Congenital Heart Disease and Pediatric Cardiology, University Hospital Schleswig Holstein, Kiel, Germany, Kiel, Germany; 2 Department of Radiology, Harvard Medical School, Women's Hospital, Boston, Boston, MA, USA

## Background

Aortic Coarctation requires a long term follow-up in order to detect late sequelae such as re-stenosis, aneurysms or arterial hypertension.

The aim of this CMR study was to measure the myocardial extracellular volume fraction (ECV) as a surrogate marker of diffuse myocardial fibrosis in patients with aortic coarctation after surgical repair in infancy.

## Methods

As a part of comprehensive CMR examination myocardial extracellular volume fraction (ECV) was calculated from pre-and post- gadolinium contrast T1 measurements of blood and myocardium (Look-Locker technique) in 25 asymptomatic patients (age:18.9±7.6) with aortic coarctation after surgical repair and 10 age-matched controls. Parameters of ventricular volumes were obtained from cine CMR. All patients had normal parameters of systolic and diastolic function by echocardiography.

## Results

Patients with aortic coarctation after surgical repair had an increased ECV compared to the control subjects 0.30±0.05; range 0.24-0.47 vs. 0.26±0.15 (range 0.28-0.23), p < 0.05. LV ejection fraction was 64±5.4% vs 60±4.2% (p=ns); indexed left ventricle end-diastolic volume was 80.7±21.4 ml/m2 vs 78.7±11.8 ml/m2 (p=ns); indexed left ventricle end-systolic volume was 32.1±2.1 ml/m2 vs 31.2±5.5 ml/m2 (p=ns); LV mass/BSA was 55.6±16.1 g/m2 vs 51.8±11.6 g/m2 (p=ns). LV mass/volume was 0.69±0.2 g/ml vs 0.67±0.2 g/ml (p=ns). Mild re-coarctation (ΔP≤10 mmHg) was present in four patients. One of them developed arterial hypertension which was controlled medically. None of the 25 patients had to undergo a reoperation in the past.

## Conclusions

Two decades after surgical repair of aortic coarctation at infancy asymptomatic patients show an increased myocardial extracellular volume fraction (ECV) indicative of adverse tissue remodeling. Our results suggest that these patients may have a higher lifetime risk for diastolic dysfunction and arrhythmias despite optimal surgical results.

## Funding

N/A.

